# Supplementary material for: Tough fiber-reinforced composite ionogels with crack resistance surpassing metals
Source: Nat Commun. 2025 Apr 29;16:4005. doi: 10.1038/s41467-025-59396-9 (PMC12041386; doi:10.1038/s41467-025-59396-9)
Supplement: Supplementary file 1 — Supplementary Information [file 41467_2025_59396_MOESM1_ESM.pdf]

Supplementary Information for

Tough Fiber-Reinforced Composite Ionogels with Crack Resistance Surpassing Metals

Xiaolin Lyu,[1] * *Kun Yu,*[1] *Haoqi Zhang,*[1] *Piaopiao Zhou,*[2,] * *Zhihao Shen*,[3] *Zhigang Zou*[1, 4,] *

[1] Key Laboratory of Advanced Materials Technologies, College of Materials Science and Engineering, Fuzhou University, Fuzhou 350108, China

[2] Department of Critical Care Medicine, Fujian Medical University Union Hospital, Fuzhou 350001, China

[3] Key Laboratory of Polymer Chemistry and Physics of Ministry of Education, College of Chemistry and Molecular Engineering, Peking University, Beijing 100871, China

[4] Eco-materials and Renewable Energy Research Center, College of Engineering and Applied Sciences, Nanjing University, Nanjing 210093, China

Table of Contents

Supplementary Figures S1-S59 ………………………………… **S2**

Supplementary Tables S1-S6 ………………………………… **S61**

Supplementary References S1-S20 ………………………………… **S68**

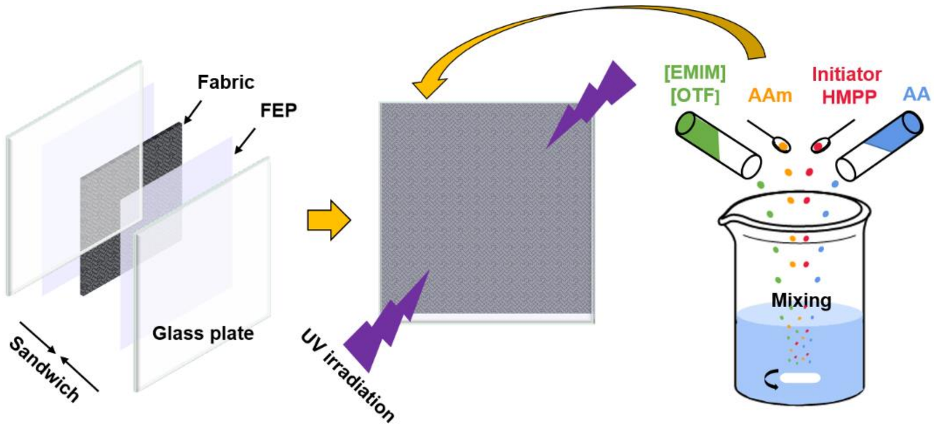

Supplementary Figure 1. Schematic of the preparation process of the FRCI. FRCI is prepared by placing the fiber fabric and precursor solution into a glass mold, followed by initiating a polymerization reaction using ultraviolet light.

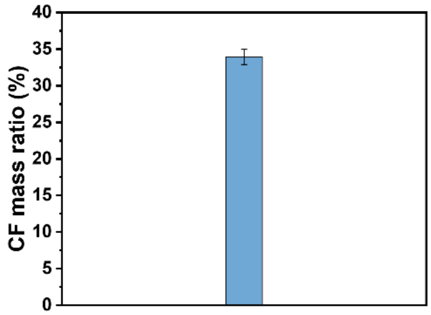

Supplementary Figure 2. CF mass ratio in the FRCI. Data are reported as their means ± SDs from *n* = 3 independent samples.

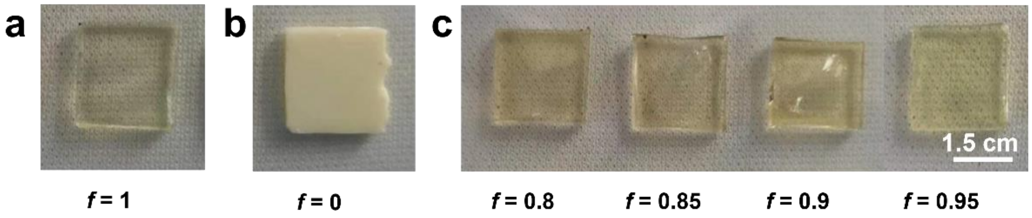

Supplementary Figure 3. Photographs of ionogels with different AA ratios. (a) Ionogel composed of PAA and [EMIM][OTf]. (b) Ionogel composed of PAAm and [EMIM][OTf]. (c) Ionogels composed of P(AA-*co*-AAm) and [EMIM][OTf] with different AA ratios and 60% IL content. The copolymer has good compatibility with the ionic liquid.

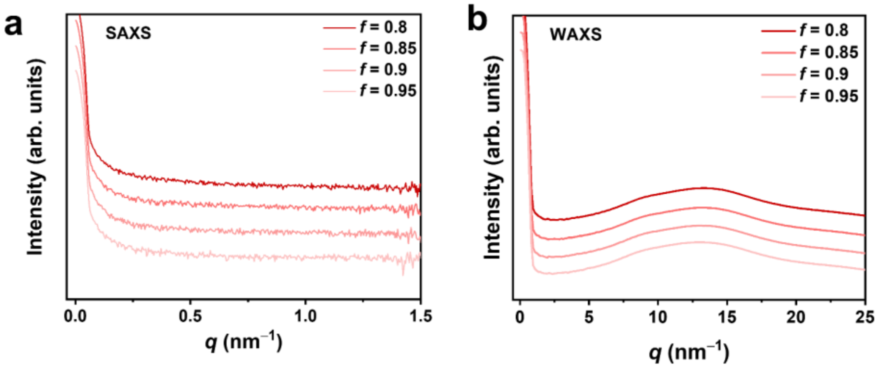

Supplementary Figure 4. SAXS (a) and WAXS (b) results of ionogels with different AA ratios and 60% IL content. The absence of peaks in the SAXS results indicates good compatibility between the polymer and ionic liquid in the ionogel. The broad peaks in the WAXS results are caused by scattering between the amorphous polymer chains.

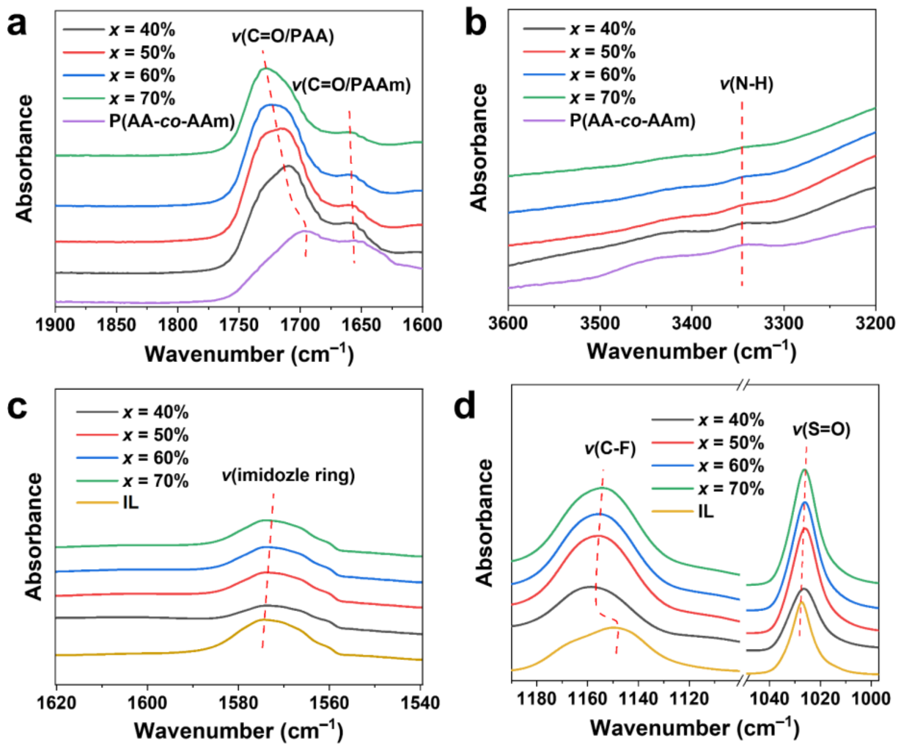

Supplementary Figure 5. Infrared results of P(AA-*co*-AAm), IL, and ionogels with the AA ratio of 0.85 and different IL contents. (a) *v*(C=O/PAA) and *v*(C=O/PAAm); (b) *v*(N-H); (c) *v*(imidazole ring); (d) *v*(C-F) and *v*(S=O).

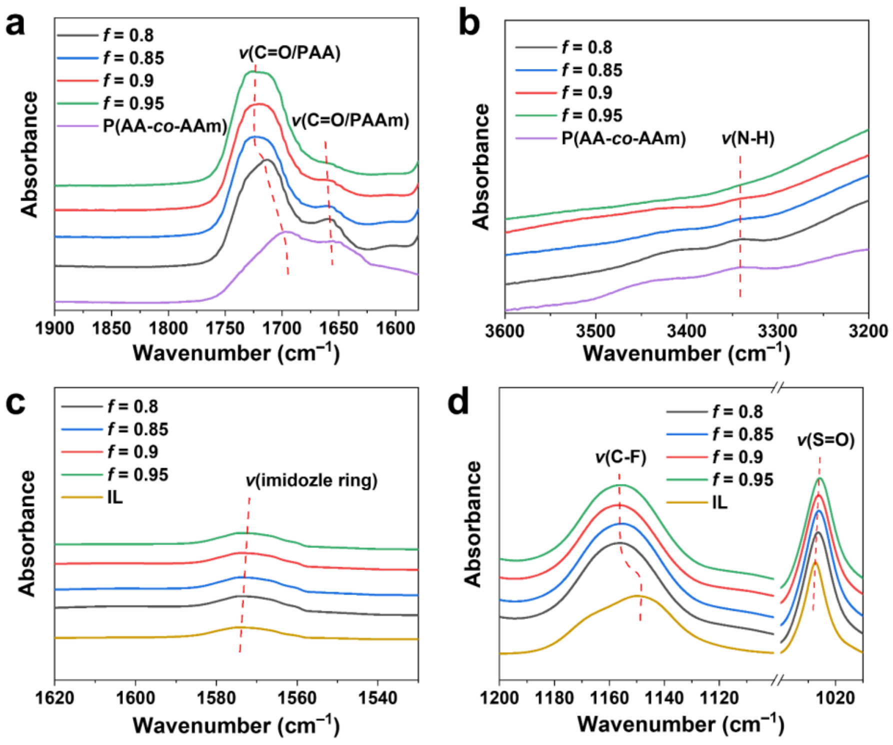

Supplementary Figure 6. Infrared results of P(AA-*co*-AAm), IL, and ionogels with the IL content of 60% and different AA ratios. (a) *v*(C=O/PAA) and *v*(C=O/PAAm); (b) *v*(N-H); (c) *v*(imidazole ring); (d) *v*(C-F) and *v*(S=O).

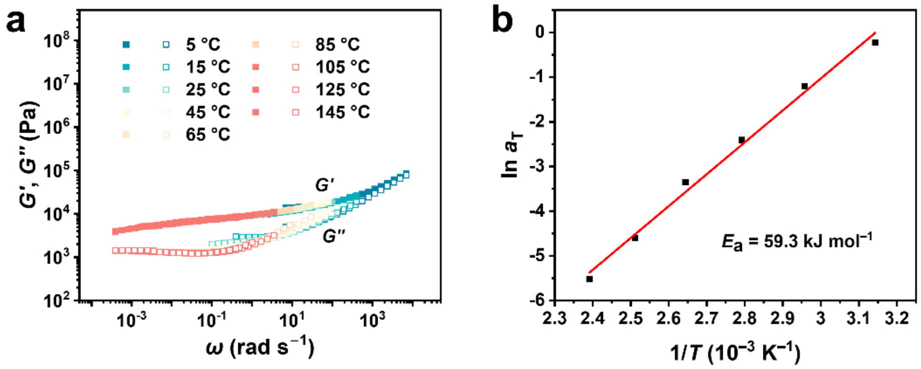

Supplementary Figure 7. Rheological results of the ionogel. (a) Rheological master curve of IG-0.85-60% from 5 ºC to 145 ºC. (b) Relationship between the shift factor and temperature.

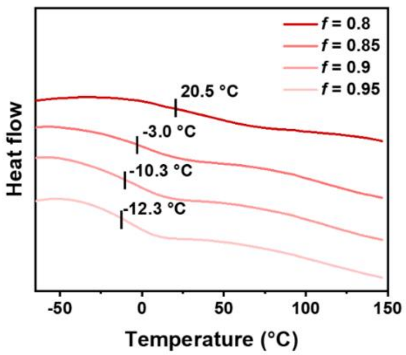

Supplementary Figure 8. DSC results of ionogels with different AA ratios. T_g increases with increasing the AAm ratio.

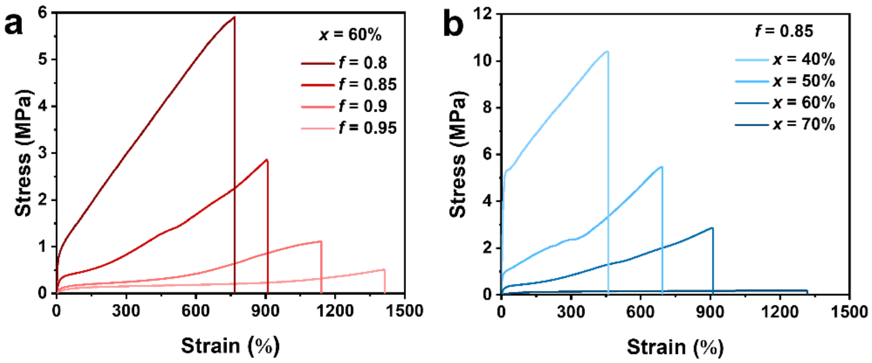

Supplementary Figure 9. Tensile stress-strain curves of ionogels with different AA ratios (a) and IL contents (b). The mechanical properties of ionogels can be tuned over a wide range by varying the AA ratio and IL content. When the AA ratio or IL content is high, the ionogel is soft and stretchable. While the AA ratio or IL content is low, the ionogel is strong and tough.

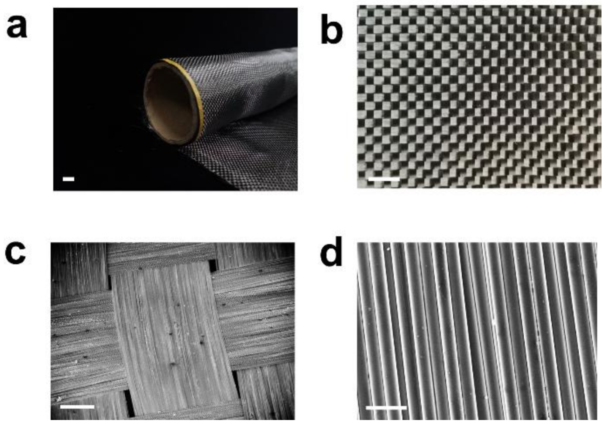

Supplementary Figure 10. Images of plain weave CF with fiber bundles containing thousands of individual fibers. (a) Photograph of the CF fabric. Scale bar: 1 cm. (b) Optical microscope image of the plain weave structure. Scale bar: 10 mm. (c-d) SEM micrographs of the fiber bundle (c) and individual fibers (d). The scale bars in (c) and (d) are 500 μm and 20 μm, respectively. The parameters and mechanical properties of the CF fabric are summarized in Supplementary Tables 1 and 2.

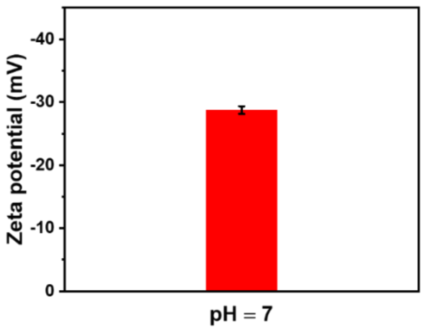

Supplementary Figure 11. Zeta potential of the CF fabric. The negative potential reveals that the surface of the fabric contains massive negative charges. Data are reported as their means ± SDs from n = 3 independent samples.

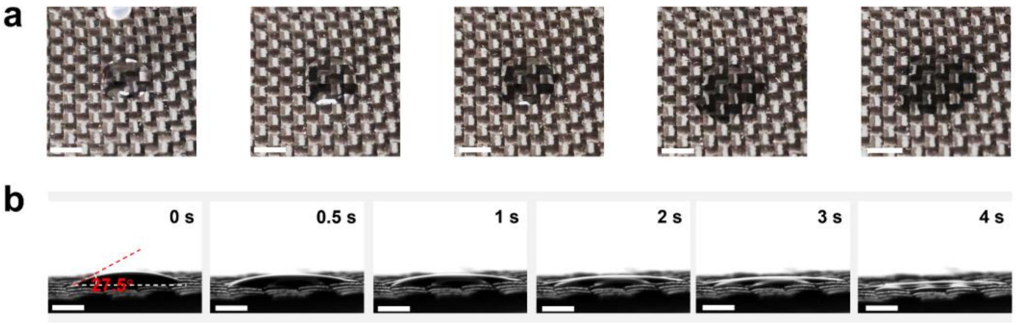

Supplementary Figure 12. Wettability test of the precursor solution on CF. Photographs (a) and contact angle (b) of the precursor solution spreading on the CF fabric within 4 seconds. Scale bars in (a) and (b) are 5 mm and 2 mm, respectively.

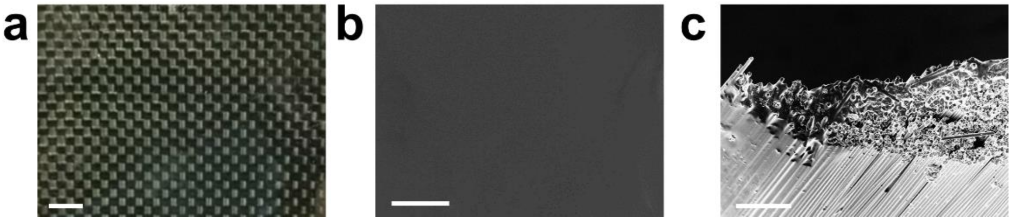

Supplementary Figure 13. Images of the FRCI composed of the ionogel and the CF fabric. (a) Optical microscope image of the FRCI. Scale bar: 10 mm. (b-c) SEM micrographs of the surface (b) and cross-section (c) of the FRCI. Scale bar: 100 μm. The surface and interior of the FRCI are covered and filled with the ionogel. Adjacent fibers are tightly bound together due to ionogel adhesion.

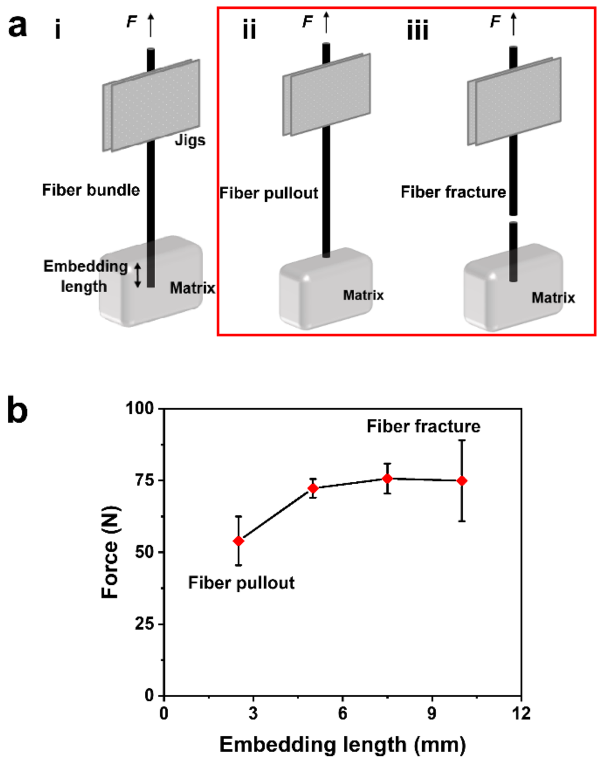

Supplementary Figure 14. Interfacial adhesion between the fiber bundle and ionogel. (a) Schematic of the fiber pullout test to estimate the interfacial bonding strength between the ionogel and CF fabric by changing the embedding length of the fiber bundle. With increasing the embedding length, the fiber bundle will change from fiber pullout to fiber fracture. (b) Adhesion force-embedding length dependence of the fiber bundle in IG-0.85-60%. The critical length of the transition between fiber fracture and fiber pullout is about 2.5 mm. Therefore, the interfacial bonding strength (τ_s) between the CF bundle and ionogel can be estimated to be 4.8 MPa, which is calculated by the equation $\tau_s = F/A$, where F (54 N) and A (11.25 mm^2) are the force when the fiber pullout occurs and the surface area of the fiber bundle that is embedded in the ionogel when the embedding length is 2.5 mm, respectively. Data in b are reported as their means ± SDs from $n = 3$ independent samples.

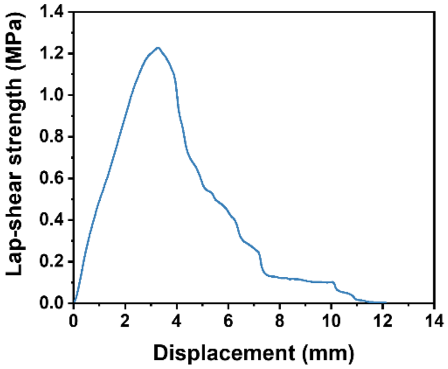

Supplementary Figure 15. Adhesion between the CF fabric and the ionogel. The lap-shear test result indicates that the adhesion strength is 1.23 MPa.

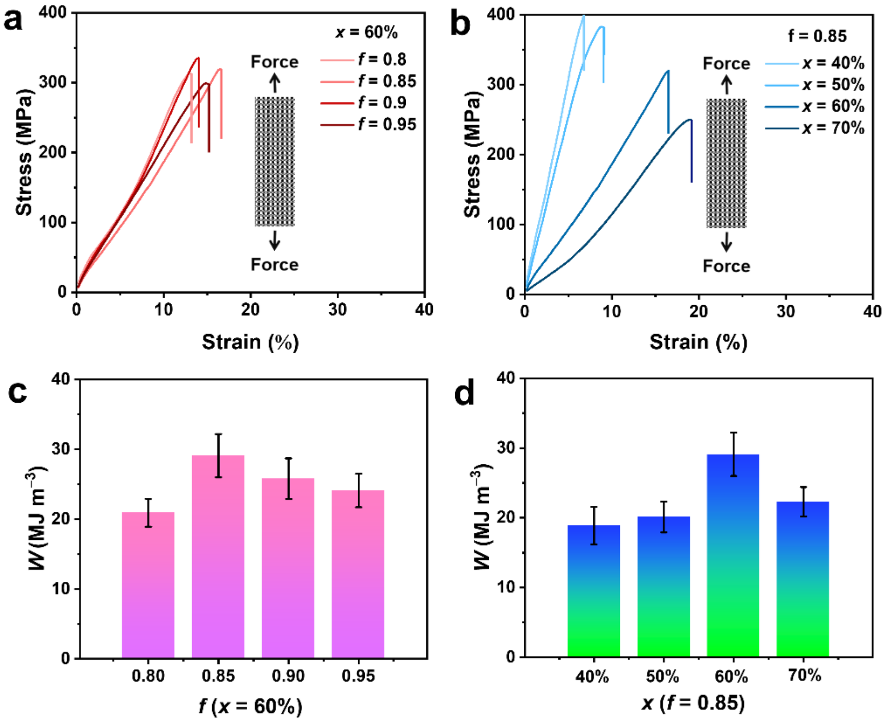

Supplementary Figure 16. Mechanical properties of FRCIs. (a-b) Tensile stress-strain curves of FRCIs with different AA ratios (a) and IL contents (b). (c-d) Work of extension of FRCIs with different AA ratios (c) and IL contents (d). All FRCIs exhibit higher strength and work of extension than the CF fabric and ionogels. Data in c and d are reported as their means ± SDs from n = 3 independent samples.

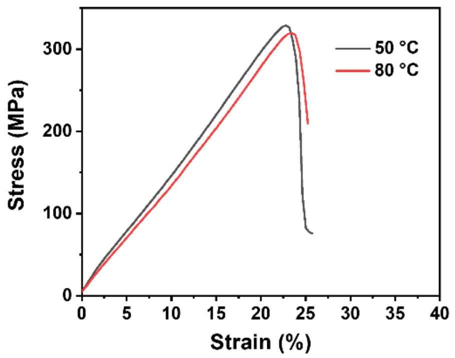

Supplementary Figure 17. Stress-strain curves of FRCI-0.85-60% at 50 °C and 80 °C. Compared to room temperature, the increase in temperature results in a slight decrease in the modulus and a slight increase in the stretchability.

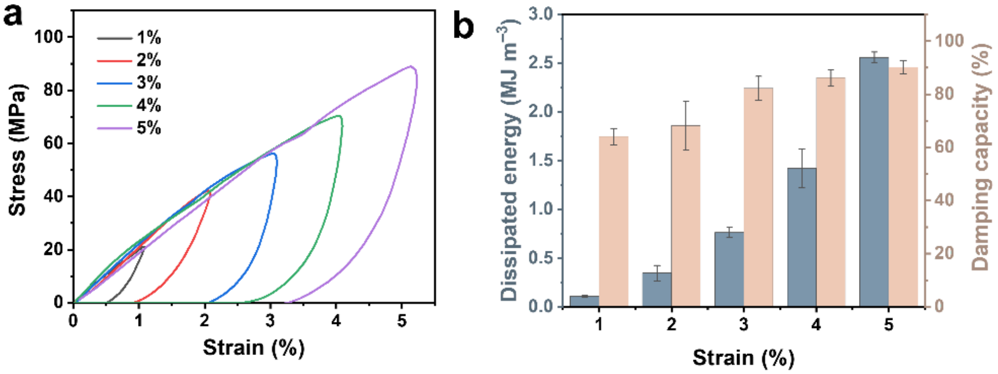

Supplementary Figure 18. Cyclic loading-unloading process and dissipated energy of FRCI. (a) Cyclic stress-strain curves of FRCI at different strains; (b) Corresponding dissipated energy and damping capacity at different strains. Data in b are reported as their means ± SDs from n = 3 independent samples.

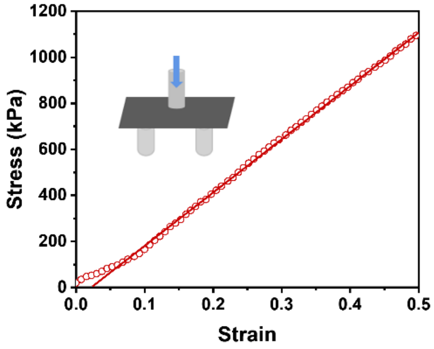

Supplementary Figure 19. Bending test of FRCI-0.85-60%. The three-point bending test shows that the bending stress increases with the increase of strain.

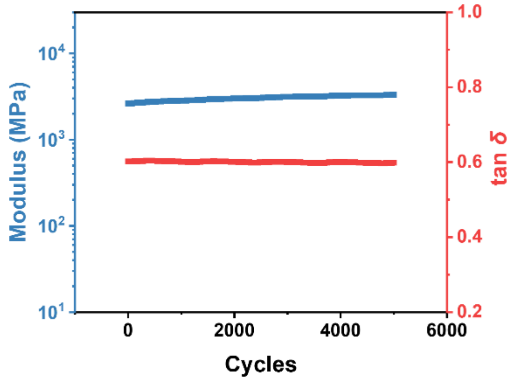

Supplementary Figure 20. Mechanical stability of FRCI. DMA results reveal that modulus and tan δ of the FRCI during cyclic stretching remain stable.

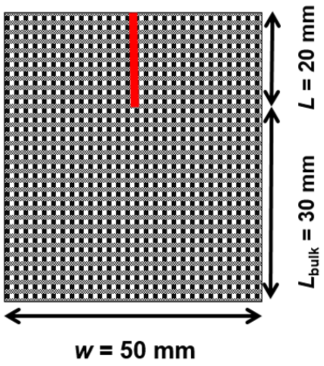

Supplementary Figure 21. Schematic of the sample shape in the trouser tearing test. The initial notch length L is fixed at 20 mm, while the projected crack length L_{bulk} is $w/2+5$ mm where w is the width of the sample. The width is set as 50 mm to ensure that the tearing behavior is dominated by fiber fracture.

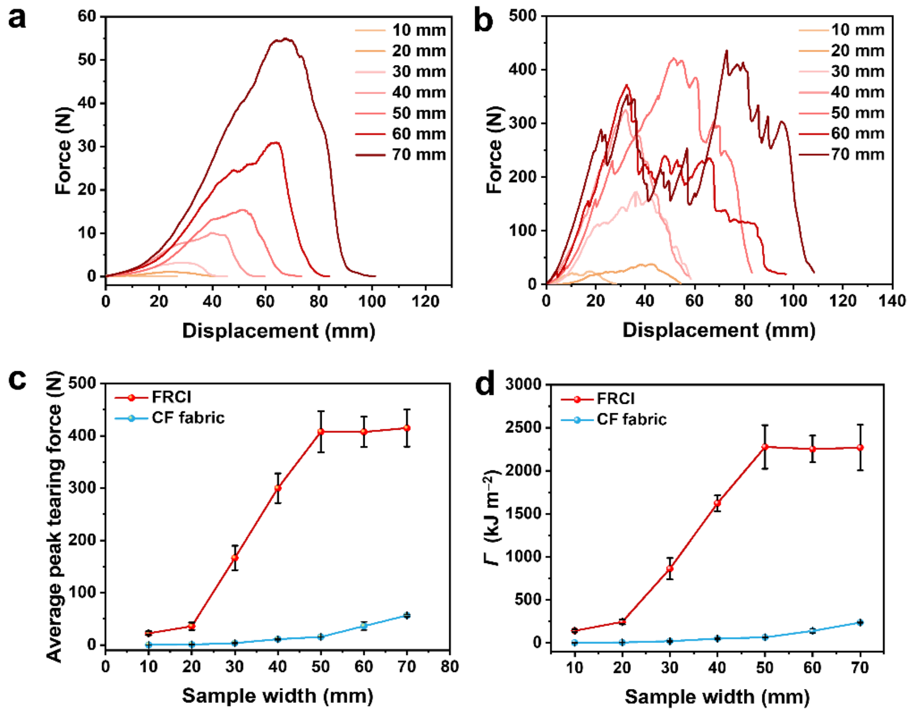

Supplementary Figure 22. Tearing behavior of FRCIs. (a-b) Force-displacement curves of the CF fabric (a) and FRCI-0.85-60% (b) with changing the sample width. (c-d) Average peak tearing force (c) and tearing toughness (d) of the CF fabric and FRCI-0.85-60% with increasing the sample width. The toughness of the FRCI increases with increasing the sample width, saturating at w = 50 mm of 2278 kJ m^{-2}. In comparison, the toughness of the pure CF fabric is much lower than that of the FRCI. Data in c and d are reported as their means ± SDs from n = 3 independent samples.

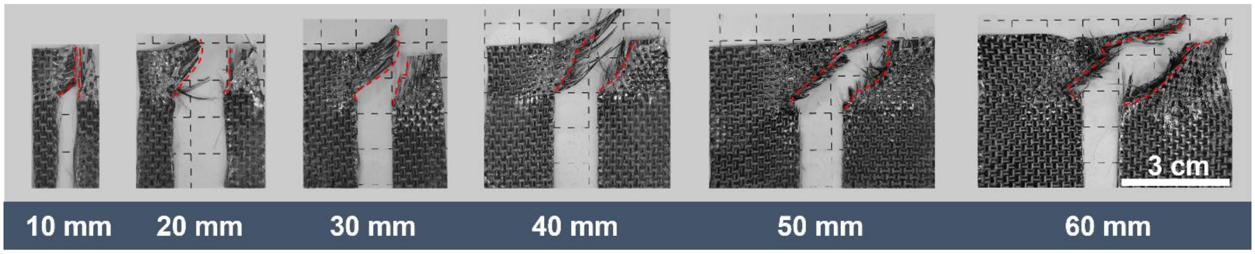

Supplementary Figure 23. Photographs of FRCI-0.85-60% with different sample widths after trouser tearing. With increasing the sample width, the tearing behavior transforms from the fiber pullout to the fiber fracture.

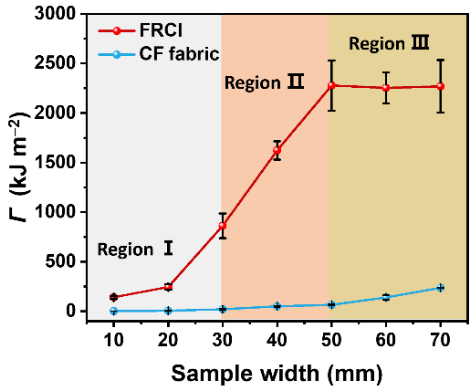

Supplementary Figure 24. Tearing behavior of the FRCI with increasing the sample width can be divided into three regions. Transverse fiber bundles are pulled out in Region I ($w \leqslant 30$ mm), indicating the total shear stress applied to the fiber bundle was much lower than its fracture strength. Fiber fracture starts at $w = 40$ mm, and fiber pullout and fiber fracture coexist in Region II. Fiber fracture dominates in Region III ($w \geqslant 50$ mm), where the tearing toughness reaches saturation at $w = 50$ mm (2278 kJ m^{-2}) and is independent of size. Data are reported as their means ± SDs from $n = 3$ independent samples.

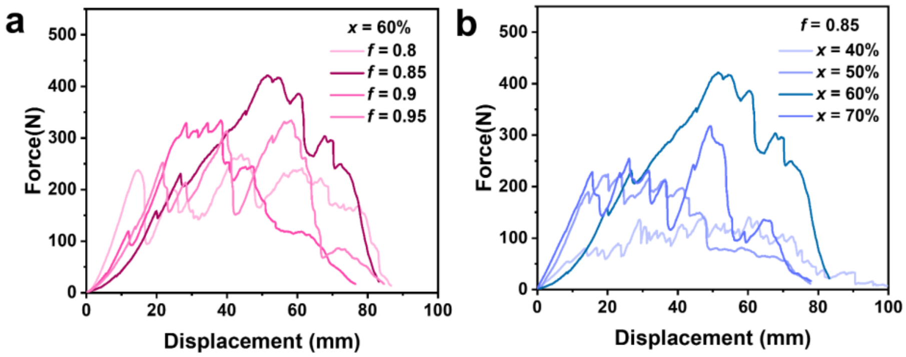

Supplementary Figure 25. Trouser tearing of FRCIs. (a) Force-displacement curves of FRCI with different AA ratios. (b) Force-displacement curves of FRCI with different IL contents.

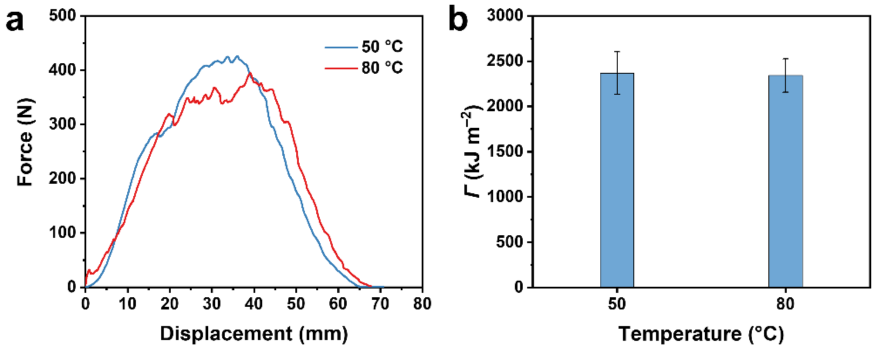

Supplementary Figure 26. Tearing curves (a) and tearing toughness (b) of FRCI-0.85-60% at 50 °C and 80 °C. Data in b are reported as their means ± SDs from $n = 3$ independent samples.

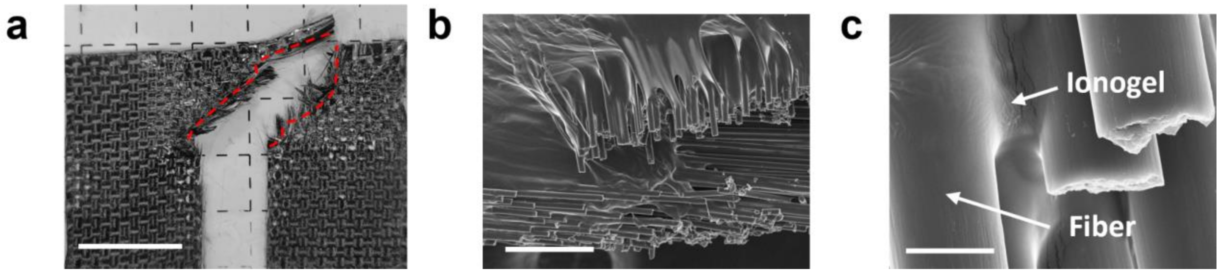

Supplementary Figure 27. Interfacial adhesion demonstration of the FRCI. Photographs (a) and SEM micrographs (b-c) of the FRCI after tearing. Scale bar: 2 cm in (a), 100 μm in (b), and 5 μm in (c). The precursor solution can penetrate into the fiber bundle to form a strong adhesion interface. Meanwhile, the adhesion interface between ionogel and the CF fabric remains unchanged even after the tearing. Their strong bonding can enable CF to fracture and ionogel to severely deform for dissipating a large amount of energy.

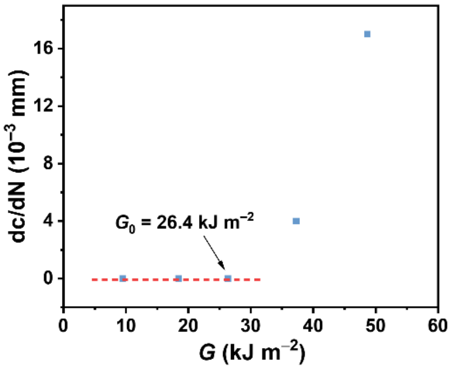

Supplementary Figure 28. Fatigue test of FRCI. Crack extension per cycle dc/dN versus applied energy release rate G for the FRCI. The fatigue threshold is 26.4 kJ m^{-2}.

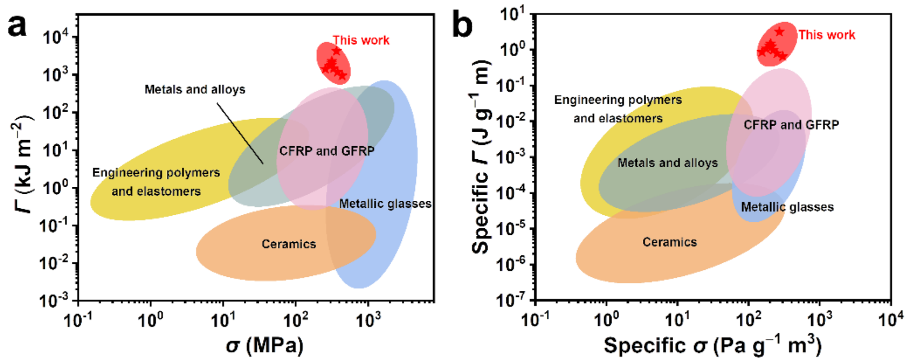

Supplementary Figure 29. **Comparison of FRCIs in this work with first-class industrial materials.** (a) Comparison of tearing toughness and tensile strength. (b) Comparison of specific tearing toughness and specific tensile strength.

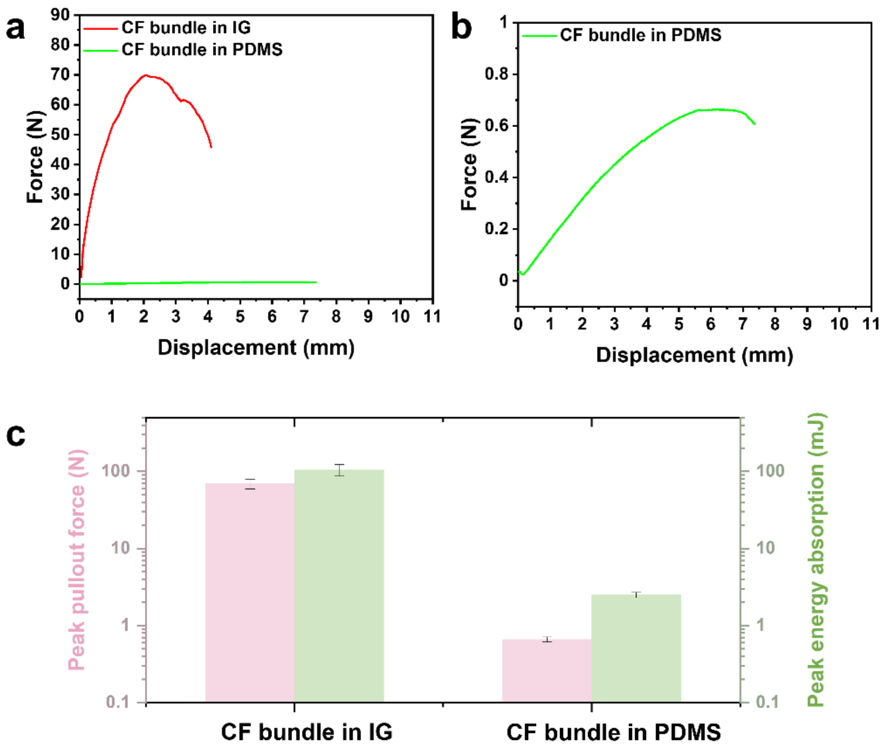

Supplementary Figure 30. Comparison of interfacial adhesion between FRCI and PDMS/CF. (a) Force-displacement curves of the CF bundle embedding in IG-0.85-60% and PDMS through the pullout test. (b) Enlarged view of the force-displacement curve of the CF bundle embedding in PDMS in Supplementary Figure 30a. (c) Peak pullout force and peak energy absorption of the CF bundle embedding in IG and PDMS. The interfacial bonding between the CF bundle and the IG is much stronger than that between the CF bundle and PDMS. Therefore, the pullout force and energy required for pulling out the fiber bundle in IG are much larger. Data in c are reported as their means ± SDs from n = 3 independent samples.

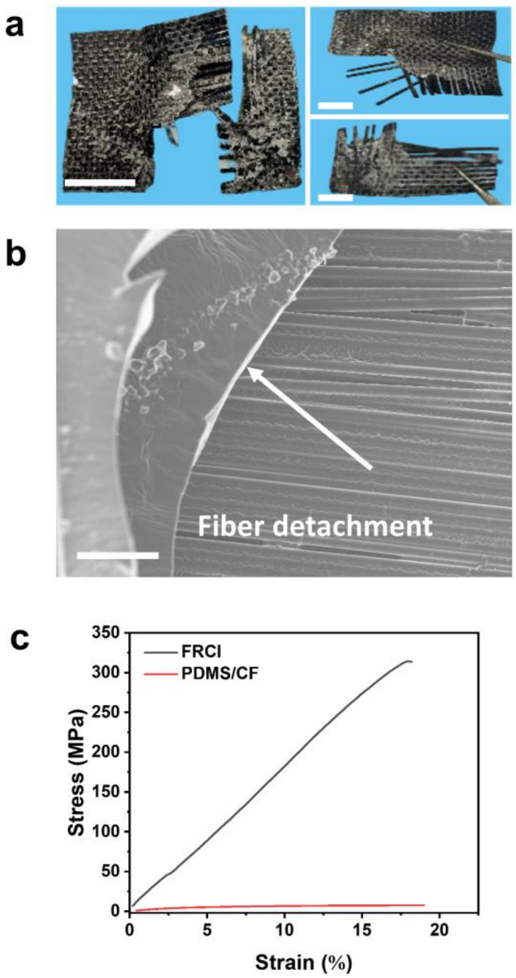

Supplementary Figure 31. Interfacial adhesion demonstration and mechanical properties of the FRCI. (a-b) Photographs (a) and SEM micrograph (b) of PDMS/CF after tearing. (c) Tensile stress-strain curves of FRCI-0.85-60% and PDMS/CF. Scale bar: 2 cm in (a) and 50 μm in (b). The weak adhesion interface between PDMS and the CF fabric leads to the fiber pullout phenomenon after the tearing. Meanwhile, the tensile strength of PDMS/CF is much lower than that of the FRCI.

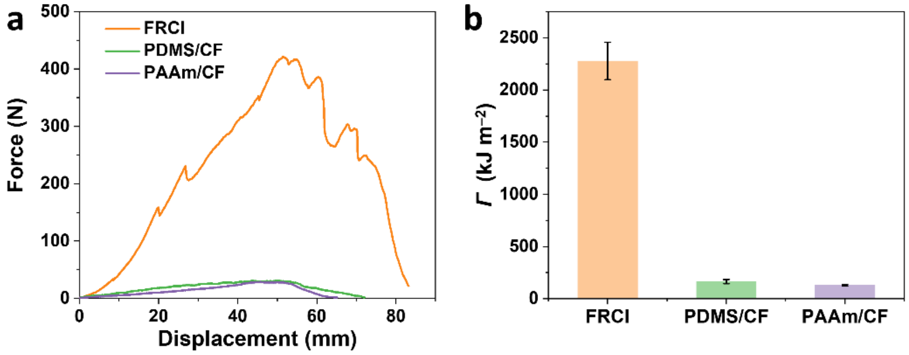

Supplementary Figure 32. Comparison of tearing toughness. Force-displacement curves (a) and toughness (b) of FRCI-0.85-60%, PDMS/CF, and PAAm hydrogel/CF after tearing. The FRCI exhibits extraordinary toughness compared with other composite systems. Data in b are reported as their means ± SDs from $n = 3$ independent samples.

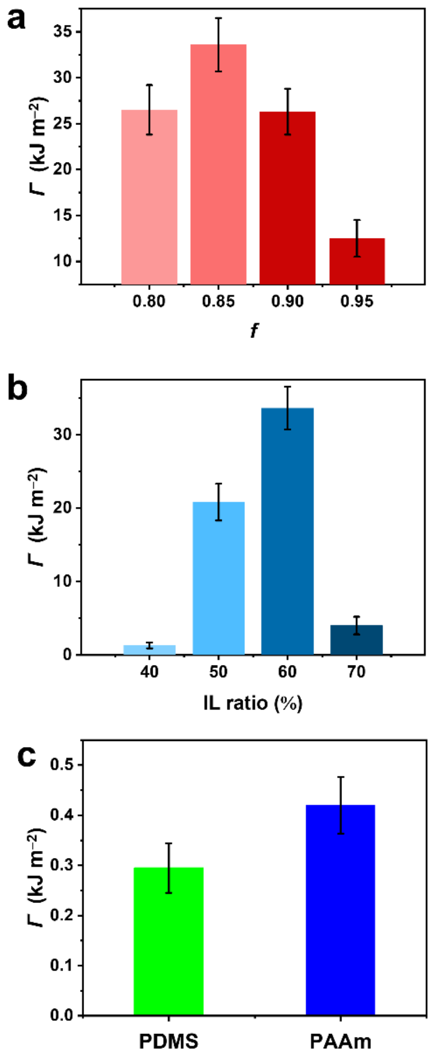

Supplementary Figure 33. Toughness of ionogels with different AA ratios (a), ionogels with different IL contents (b), PDMS, and PAAm hydrogel (c). The sample width was made into 50 mm as shown in Supplementary Figure 21. The toughnesses of ionogels, PDMS, and PAAm hydrogel were used as *x*-axis data in Figure 3a. Data in a, b, and c are reported as their means ± SDs from $n = 3$ independent samples.

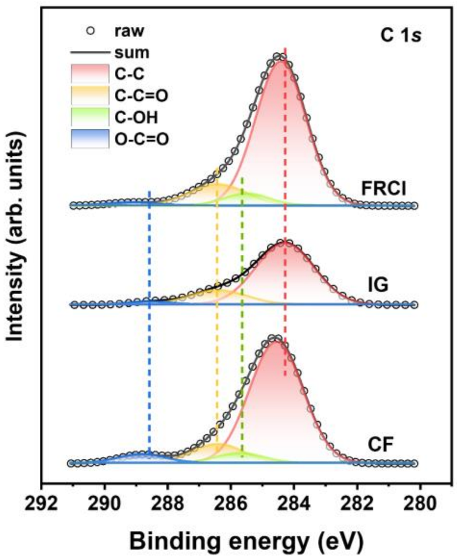

Supplementary Figure 34. XPS C 1*s* spectra of the FRCI, ionogel, and CF fabric. The shift of C 1*s* peaks of O-C=O and C-OH reveals that there is a tight bond between the ionogel and CF fabric.

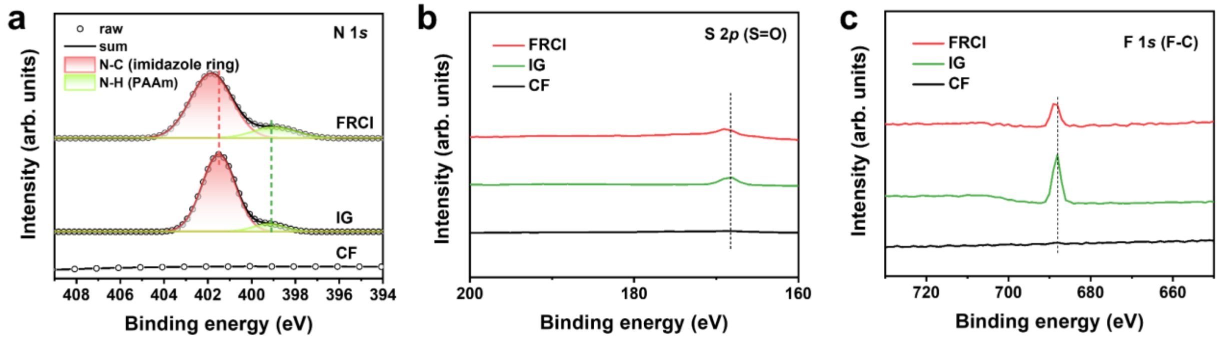

Supplementary Figure 35. XPS spectra of the FRCI, ionogel and CF fabric. (a) N 1*s* spectra. (b) S 2*p* spectra. (c) F 1*s* spectra.

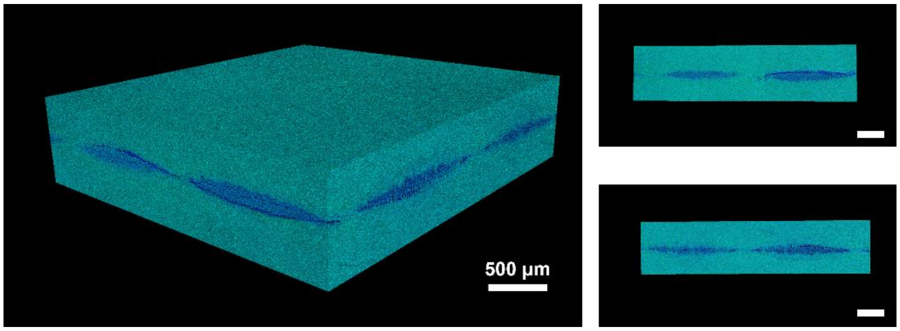

Supplementary Figure 36. CT results of the FRCI. The images reveal that the ionogel within the FRCI is tightly bound to the carbon fibers. Scale bar: 500 μm.

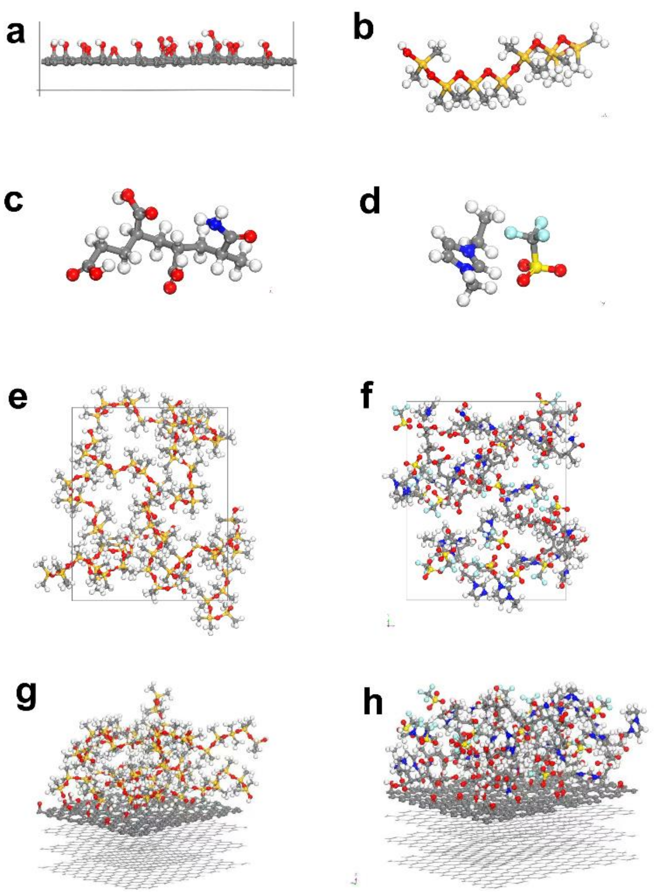

Supplementary Figure 37. Molecular model of DFT calculations. (a) Surface structure of CF with hydroxy and carboxyl groups on the surface. (b-d) molecular models of PDMS (b), P(AA-*co*-AAM) (c), and [EMIM][OTf] (d). (e-f) Amorphous models of PDMS (e) and ionogel (f) with the cell parameter the same as that of CF. (g-h) Covering the PDMS (g) and ionogel (h) amorphous cells on the surface of CF using a build layer tool with an initial distance of 4 Å.

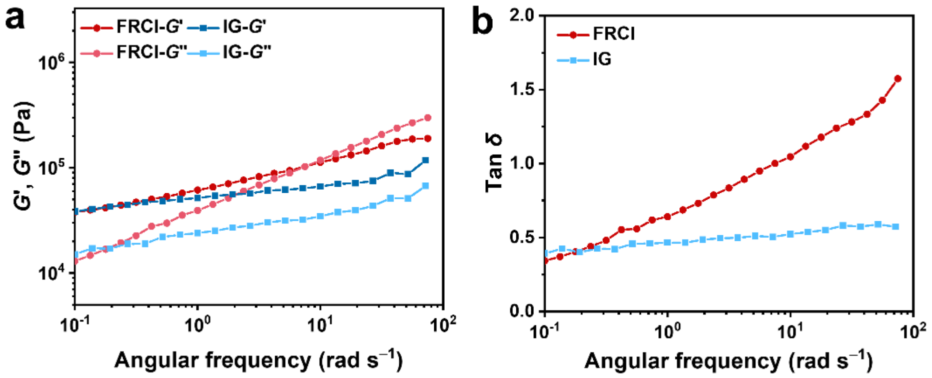

Supplementary Figure 38. Rheological results of the FRCI and ionogel. Frequency-sweeping storage modulus, loss modulus (a), and loss factor (b) of the FRCI and ionogel. The faster increase in the loss modulus of the FRCI with frequency indicates a strong interaction between the ionogel and CF, which can increase the damping capacity of the FRCI.

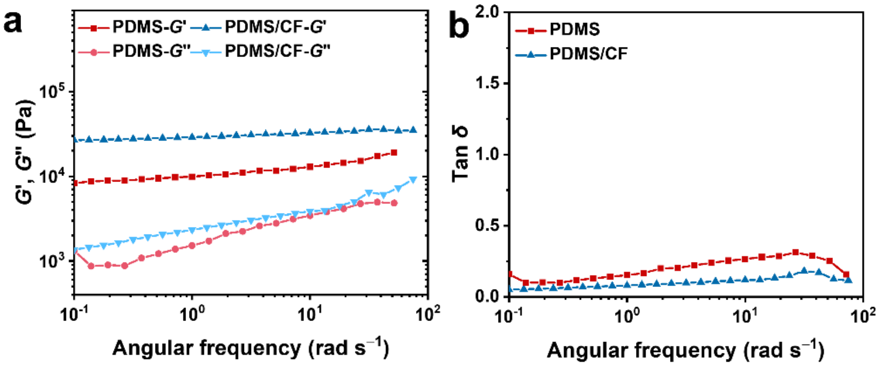

Supplementary Figure 39. Rheological results of PDMS and PDMS/CF. Frequency-sweeping storage modulus, loss modulus (a), and loss factor (b) of PDMS and PDMS/CF. The rheological results of PDMS/CF show slight changes compared to PDMS, indicating a weak interaction between PDMS and CF.

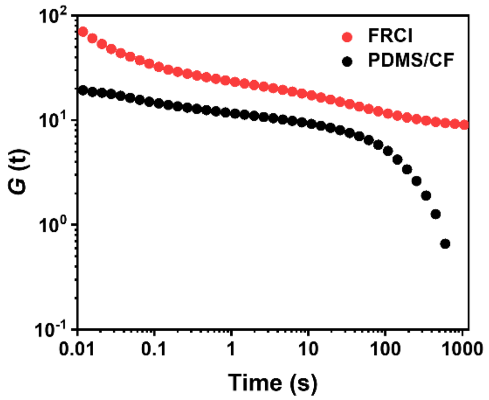

Supplementary Figure 40. Stress-relaxation behavior of the FRCI and PDMS/CF. The long relaxation time of the FRCI reveals its high structural stability.

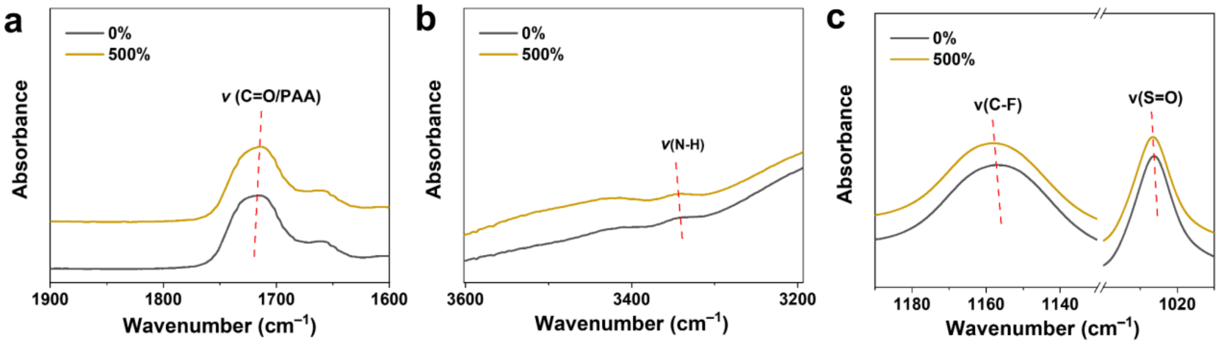

Supplementary Figure 41. Infrared results of the ionogel at 0% and 500% strain. (a) *v*(C=O/PAA); (b) *v*(N-H); (c) *v*(C-F) and *v*(S=O).

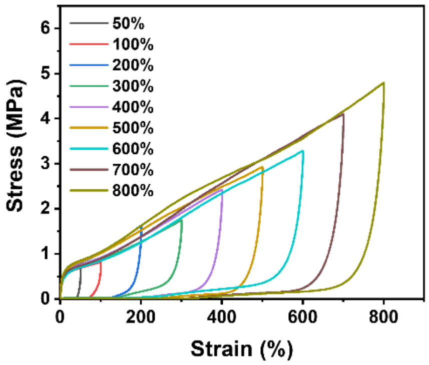

Supplementary Figure 42. Cyclic stress-strain curves of the ionogel at different strains. The dissipated energy increases with increasing the strain.

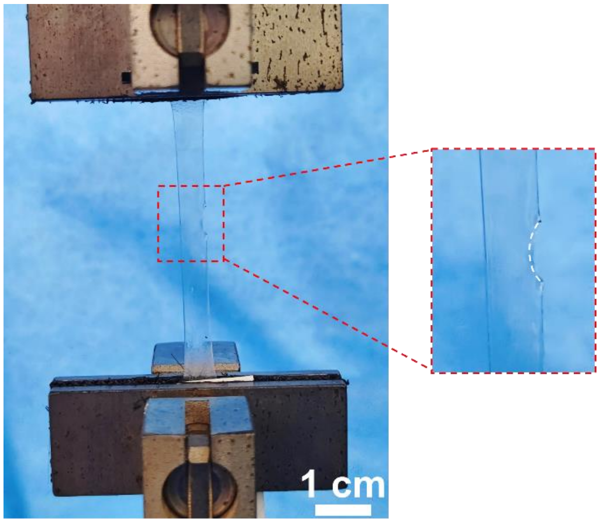

Supplementary Figure 43. Photograph of stretching the ionogel having a crack. The ionogel can blunt crack propagation and inhibit the further spread of defects,

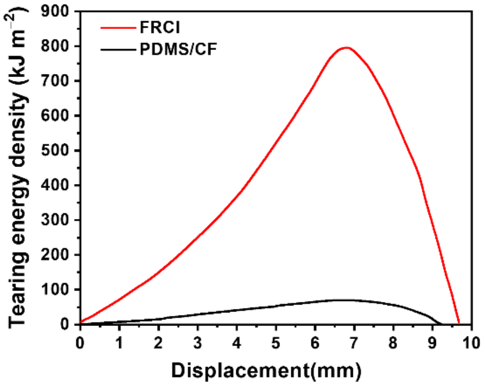

Supplementary Figure 44. Simulated tearing energy density of the FRCI and PDMS/CF. Finite-element modeling reveals that the tearing energy density of FRCI is much higher than that of PDMS/CF.

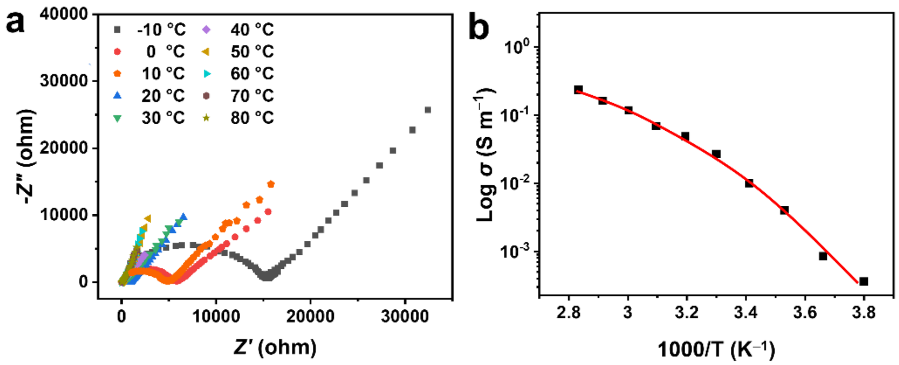

Supplementary Figure 45. Ion-conductive properties of FRCI. (a) Impedance results of FRCI-0.85-60% from –10 to 80 ºC. (b) Vogel–Tamann–Fulcher fitting of the relationship between ionic conductivity and temperature of FRCI-0.85-60%.

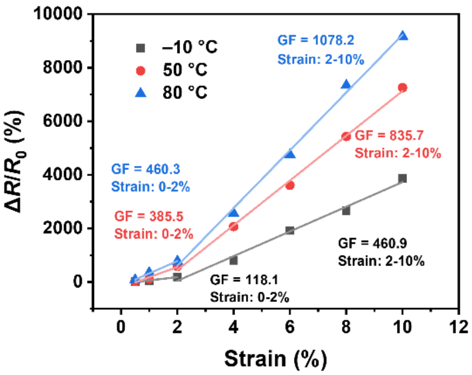

Supplementary Figure 46. GF values of FRCI-0.85-60%. FRCI has high sensitivities at different temperatures. Meanwhile, GF values increase with increasing temperature.

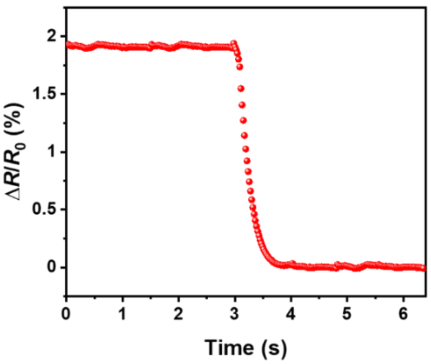

Supplementary Figure 47. Response time of the FRCI. The FRCI has a fast response time.

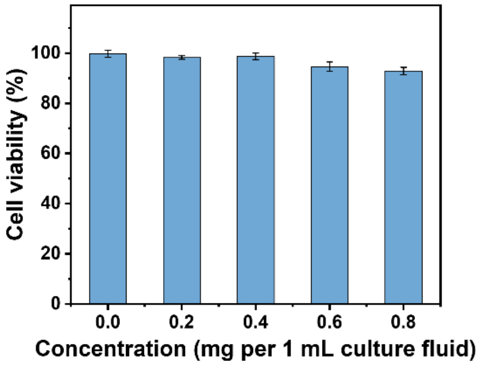

Supplementary Figure 48. Cell viability incubated with different amounts of FRCI for 24 h.

Data are reported as their means ± SDs from $n = 3$ independent samples.

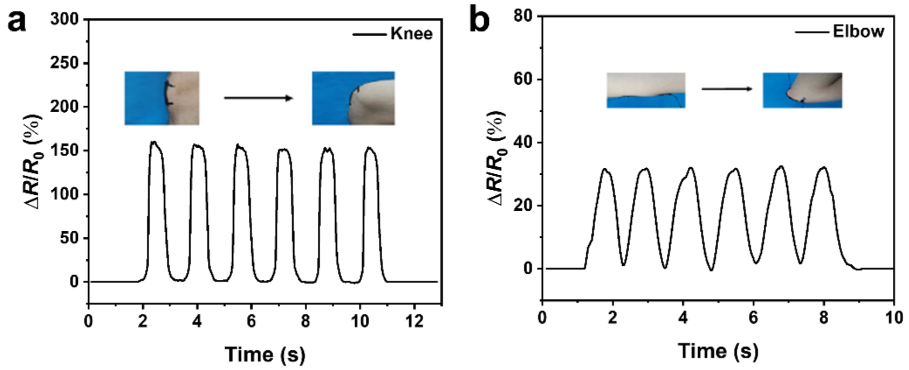

Supplementary Figure 49. FRCI attached to the joints to detect human movements. (a) Knee. (b) Elbow. The electrical signal remains stable during movements.

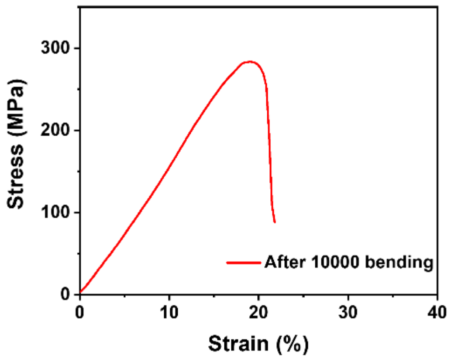

Supplementary Figure 50. Stress-strain curve of FRCI-0.85-60% after bending 10000 times. The strength and modulus only exhibit a slight decrease while the stretchability exhibits a slight increase.

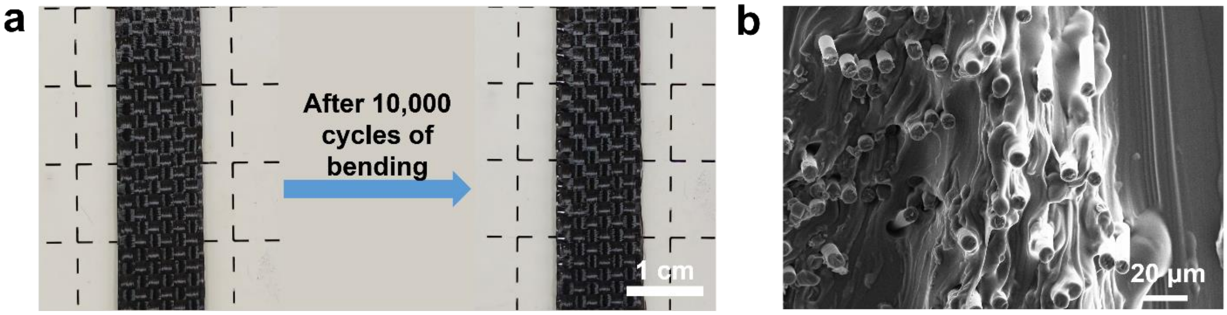

Supplementary Figure 51. Photograph (a) and SEM image (b) of the FRCI after bending 10,000 cycles. The ionogel and CF fabric remain tightly bound together without separation after cyclic bending.

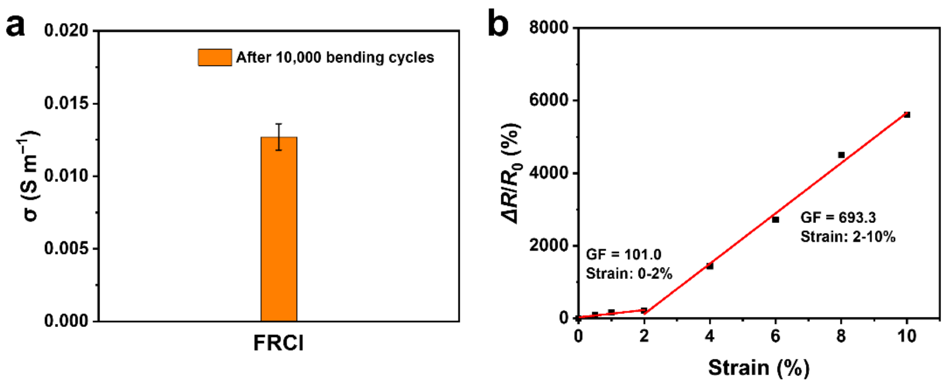

Supplementary Figure 52. Electrical properties of FRCI. (a) Room-temperature ionic conductivity of FRCI-0.85-60% after bending 10000 cycles. (b) GF values at different strains of FRCI-0.85-60% after bending 10000 cycles. Data in a are reported as their means ± SDs from n = 3 independent samples.

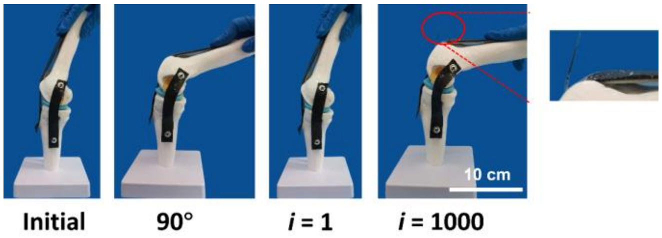

Supplementary Figure 53. Photographs of using PDMS/CF as artificial ligaments to fix artificial bones. The PDMS/CF suffers from fiber breakage after repeated bending due to its poor interfacial bonding ability and low toughness.

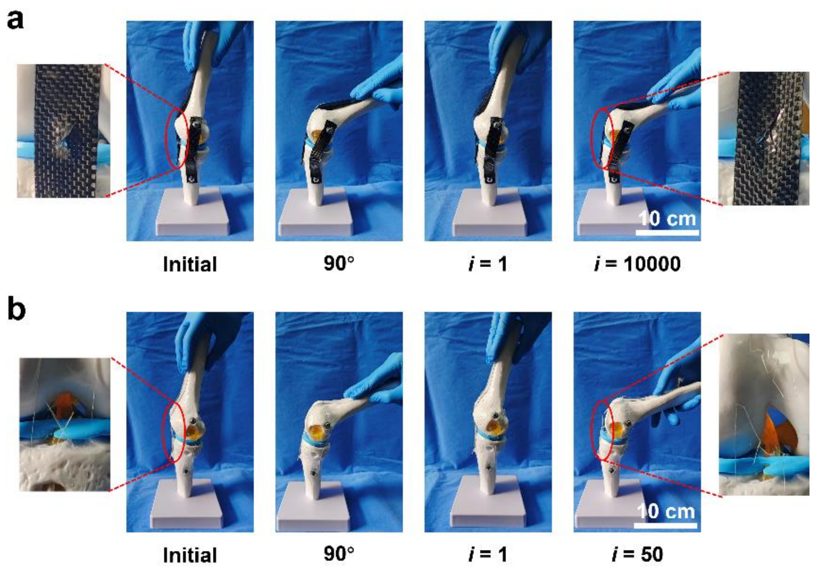

Supplementary Figure 54. Photographs of using FRCI (a) and ionogel (b) both with cracks as artificial ligaments to fix artificial bones. The FRCI remains intact after 10000 bending cycles while the ionogel suffers from breakage after only 50 bending cycles.

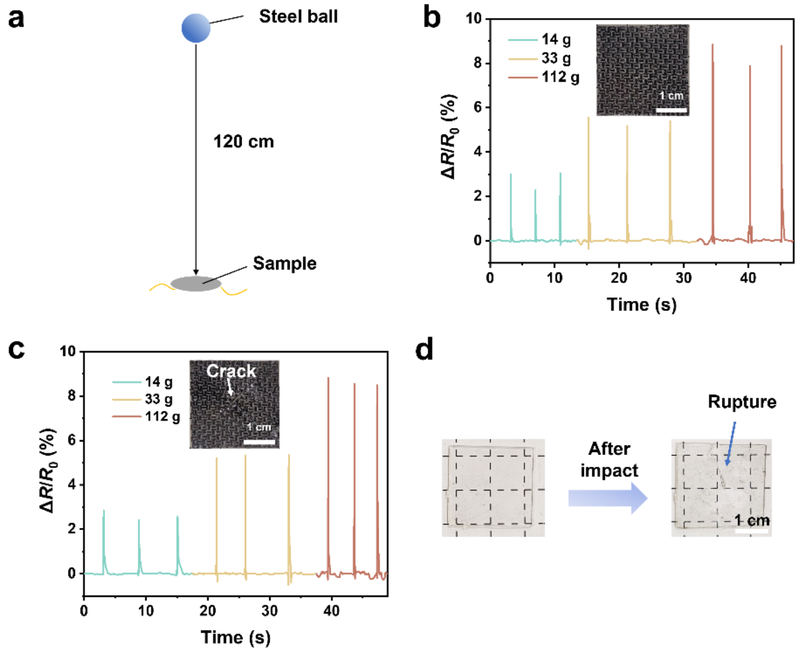

Supplementary Figure 55. Self-sensing impact protection application. (a) Schematic of the impact process. (b) Resistance signals of the impact using steel balls with different weights. (c) Resistance signals of the impact using steel balls with different weights in the presence of cracks. (d) Ionogel underwent rupture after being subjected to impact.

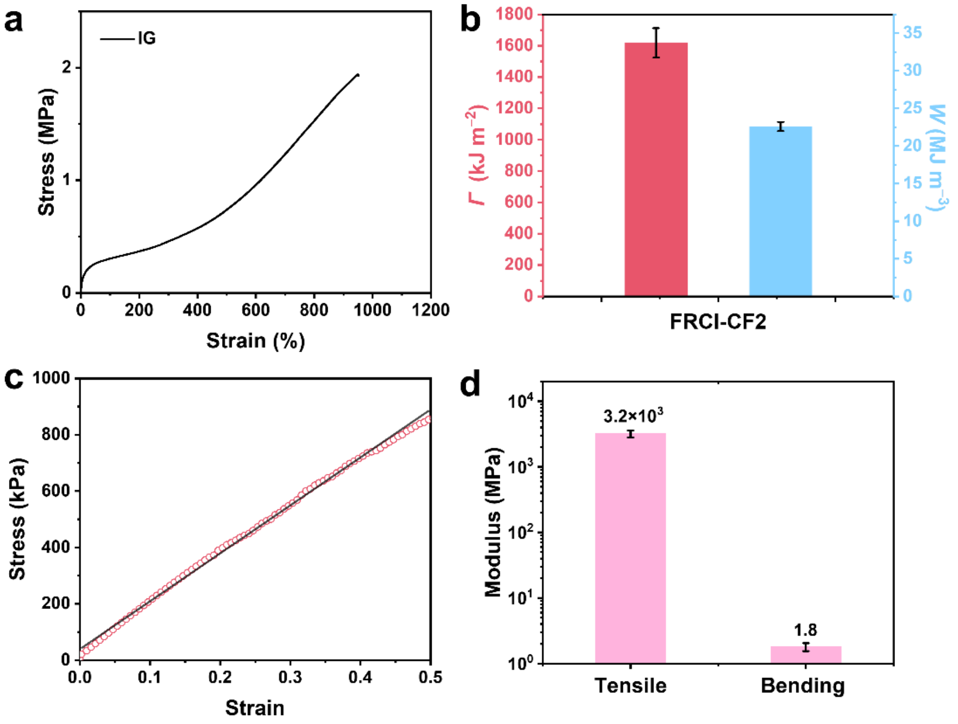

Supplementary Figure 56. Mechanical properties of IG-2 and FRCI-CF2. (a) Stress-strain curve of IG-2. (b) Toughness and work of extension of FRCI-CF2. (c) Three-point bending result of FRCI-CF2. (d) Tensile modulus and bending modulus of FRCI-CF2. Data in b and d are reported as their means ± SDs from $n = 3$ independent samples.

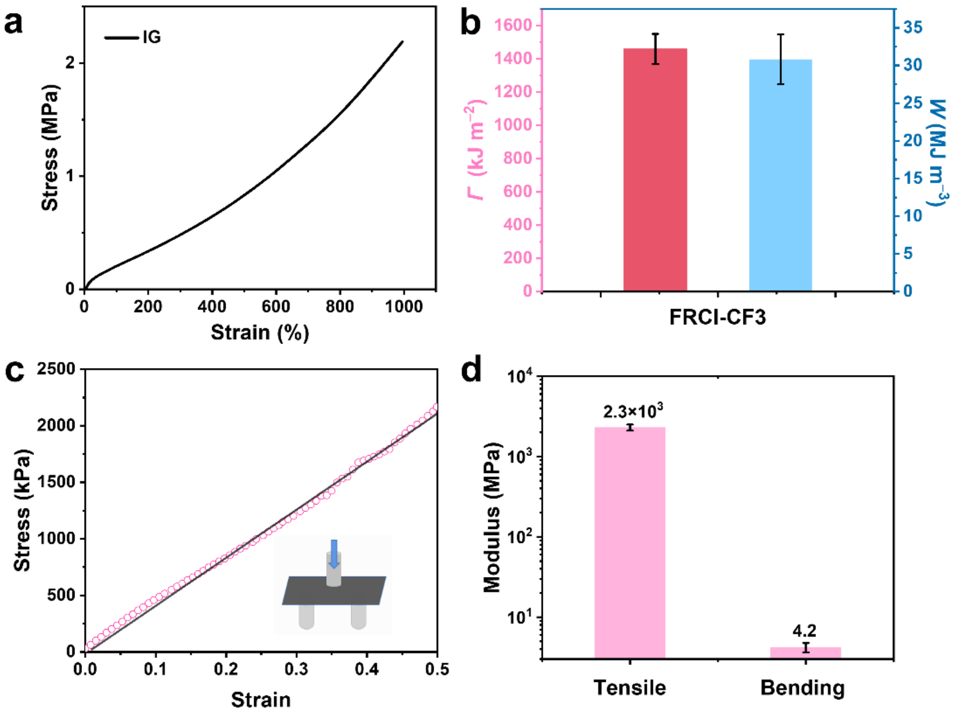

Supplementary Figure 57. Mechanical properties of IG-3 and FRCI-CF3. (a) Stress-strain curve of IG-3. (b) Toughness and work of extension of FRCI-CF3. (c) Three-point bending result of FRCI-CF3. (d) Tensile modulus and bending modulus of FRCI-CF3. Data in b and d are reported as their means ± SDs from $n = 3$ independent samples.

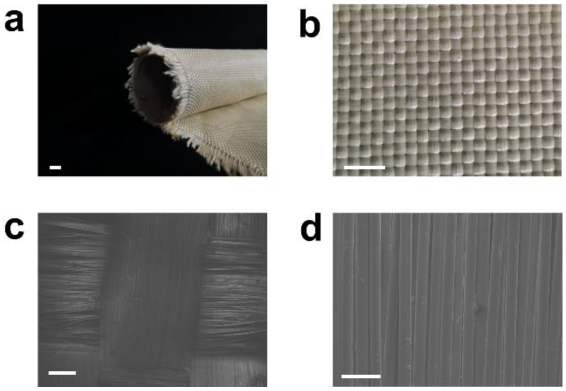

Supplementary Figure 58. Images of plain weave AF with fiber bundles containing thousands of individual fibers. (a) Photograph of the CF fabric. Scale bar: 1 cm. (b) Optical microscope image of the plain weave structure. Scale bar: 10 mm. (c-d) SEM micrographs of the fiber bundle (c) and individual fibers (d). The scale bars in (c) and (d) are 500 µm and 50 µm, respectively. The parameters and mechanical properties of the AF fabric are summarized in Tables S1 and S2.

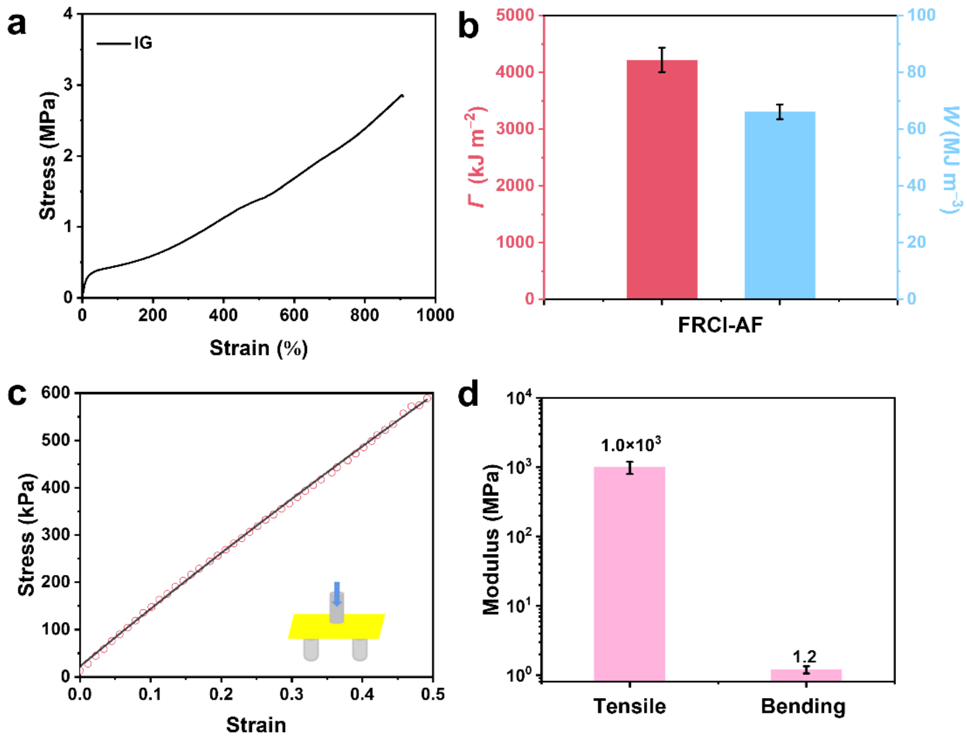

Supplementary Figure 59. Mechanical properties of FRCI-AF. (a) Stress-strain curve of IG-0.85-60%. (b) Toughness and work of extension of FRCI-AF. (c) Three-point bending result of FRCI-AF. (d) Tensile modulus and bending modulus of FRCI-AF. Data in b and d are reported as their means ± SDs from $n = 3$ independent samples.

Supplementary Table 1. Dimension parameters of the fabrics

Fabric	ρ (g cm^{-3}) a)	t (mm) a)	h (mm) a)
CF	0.92	0.25	2
AF	1.25	0.4	2

a) ρ, t, and h represent the density, fabric thickness, and width of a single bundle, respectively, of the corresponding fabric.

Supplementary Table 2. Mechanical properties of the fabrics

Sample	E (GPa) [a)]	σ (MPa) [a)]	ε (%) [a)]	W (MJ m^{-3}) [a)]
CF fabric	3.3±0.3	184±19	9.1±0.2	9.5±2.1
CF fiber bundle	12.7±1.6	334±46	7.2±0.1	18.7±3.4
AF fabric	5.6±1.3	330±41	14.2±0.4	32.1±3.4
AF fiber bundle	13.2±1.2	753±47	9.3±0.5	47.9±3.7

a) E, σ, ε, and W represent tensile modulus, fracture strength, elongation at break, and work of extension, respectively, of the corresponding fabric.

Supplementary Table 3. Mechanical properties of ionogels

Sample	*f*-*x* [b]	*E* [c] (MPa)	*σ* [c] (MPa)	*ε* [c] (%)	*W* [c] (MJ m^{-3})	*Γ* [c] (kJ m^{-2})	*Γ* / *W* [d] (mm)
IG-*f*-*x* [a]	0.8-60%	13.6±1.5	5.9±0.4	680±60	26.9±1.3	26.5±2.7	1.0±0.04
	0.85-60%	2.9±0.3	2.8±0.3	900±50	12.2±0.6	33.6±2.9	2.9±0.1
	0.9-60%	0.6±0.1	1.1±0.2	1100±100	5.8±0.3	26.3±2.5	4.5±0.18
	0.95-60%	0.3±0.1	0.5±0.1	1400±100	3.2±0.3	12.5±2.0	3.9±0.24
	0.85-40%	79.0±5.7	10.4±0.6	440±40	35.6±1.8	1.3±0.4	0.04±0.01
	0.85-50%	40.0±2.9	5.4±0.3	660±40	19.8±1.0	20.8±2.5	1.1±0.08
	0.85-70%	0.2±0.02	0.2±0.03	1300±100	2.0±0.1	4.0±1.2	2.0±0.5
IG-2 [a]	/	2.8±0.4	1.9±0.3	950±80	8.1±0.4	7.0±0.5	0.9±0.05
IG-3 [a]	/	0.5±0.1	2.2±0.2	1000±60	9.3±0.5	21.3±0.4	2.3±0.2

a) IG-*f*-*x* represents the ionogel composed of P(AA-*co*-AAM) and [EMIM][OTf], IG-2 represents the ionogel composed of P(AA-*co*-AAM) and [EMIM][ES] with the AA ratio of 0.85 and the IL content of 60 wt%, and IG-3 represents the ionogel composed of P(HEA-*co*-AAM) and [EMIM][ES] with the HEA ratio of 0.85 and the IL content of 60 wt%.

b) *f* and *x* in IG-*f*-*x* represent the mass ratio of AA in P(AA-*co*-AAM) and the mass fraction of ionic liquid in the ionogel.

c) *E*, *σ*, *ε*, *W*, and *Γ* represent tensile modulus, fracture strength, elongation at break, work of extension, and toughness, respectively, of the corresponding ionogel.

d) *Γ* / *W* represents the fractocohesive length of the ionogel, which can be used to characterize the size of the energy dissipation region.

Supplementary Table 4. Mechanical properties of FRCIs

Sample	f-x [b]	E [c] (GPa)	σ [c] (MPa)	ε [c] (%)	W [c] (MJ m^{-3})	Γ [c] (kJ m^{-2})	Γ / W [d] (mm)	ρ [c] (g cm^{-3})
FRCI-f-x [a]	0.8-60%	3.7±0.2	312±19	12.8±0.5	20.9±2.0	1803±131	86.2±2.2	1.48±0.07
	0.85-60%	3.0±0.2	315±23	17.2±0.7	29.1±3.1	2278±179	78.3±2.0	1.55±0.11
	0.9-60%	2.9±0.3	313±21	14.6±0.8	25.8±2.9	2062±146	79.9±2.8	1.60±0.08
	0.95-60%	2.5±0.2	292±20	15.0±0.8	24.1±2.4	1750±102	72.6±2.7	1.65±0.10
	0.85-40%	6.2±0.5	437±67	6.9±0.6	18.9±2.7	935±71	49.5±2.9	1.45±0.07
	0.85-50%	5.0±0.3	378±42	8.8±0.3	20.1±2.2	1174±86	58.4±2.3	1.51±0.10
	0.85-70%	1.1±0.2	256±16	18.3±1.7	22.3±2.1	1411±182	63.2±2.4	1.66±0.08
FRCI-CF2 [a]	/	3.2±0.4	257±26	15.3±0.7	22.6±0.6	1618±94	71.6±4.1	1.49±0.10
FRCI-CF3 [a]	/	2.3±0.2	332±24	17.0±1.1	30.8±3.3	1459±90	47.4±2.3	1.50±0.07
FRCI-AF [a]	/	1.0±0.2	365±24	35.0±5.0	66.1±2.6	4219±216	63.8±0.8	1.35±0.07

a) FRCI-*f*-*x* is composed of the CF fabric and IG-*f*-*x*. FRCI-CF2 is composed of the CF fabric and IG-2. FRCI-CF3 is composed of the CF fabric and IG-3. FRCI-AF is composed of the AF fabric and IG-0.85-60%.

b) *f* and *x* in FRCI represent the mass ratio of AA in P(AA-*co*-AAM) and the mass fraction of ionic liquid in the ionogel.

c) E, σ, ε, W, Γ and ρ represent tensile modulus, fracture strength, elongation at break, work of extension, toughness, and density, respectively, of the corresponding FRCI.

d) Γ / W represents the fractocohesive length of the FRCI, which can be used to characterize the size of the energy dissipation region.

Supplementary Table 5. Mechanical properties of FRCIs and recently reported gel materials. The data of gel materials were extracted from the literature.

Ref.	Material	Structure/method	*E* (MPa)	*σ* (MPa)	*Γ* (kJ m^{-2})	*W* (MJ m^{-3})	*Γ*/*W* (mm)
**This work**	Ionogel	Interfacial locking	3700	312	1803	20.9	86.2
			3000	315	2278	29.1	78.3
			2900	313	2062	25.8	79.9
			2500	292	1750	24.1	72.6
			6200	437	935	18.9	49.5
			5000	378	1174	20.1	58.4
			1100	256	1411	22.3	63.2
			3200	257	1618	22.6	71.6
			2300	332	1459	30.8	47.4
			1000	365	4219	66.1	63.8
**S1**	ionogel	Phase separation	50	12.6	24	48	0.50
			20	9	15	38	0.39
			10	6	10	30	0.33
**S2**	ionogel	Supramolecular	60	37.5	400	690	0.58
			50	53.5	520	1077	0.48
			47.5	63.2	500	1947	0.26
			45	52.8	400	1610	0.25
**S3**	ionogel	Supramolecular	55	8.8	170	80	2.13
			20	5.5	200	60	3.33
			6	4	50	80	0.63
	ionogel	Supramolecular	30	3.5	6	5	1.20
			70	8	20	15	1.33

**S4**			100<br>180	12<br>13.5	23<br>45	22<br>30	1.05<br>1.50
**S5**	ionogel	Composite	0.75<br>0.5<br>0.25<br>0.1	0.18<br>0.28<br>0.33<br>0.3	15<br>38<br>20<br>10	2.5<br>4.6<br>2.6<br>3	6.00<br>8.26<br>7.69<br>3.33
**S6**	ionogel	Composite	1	1	85	17	5.00
**S7**	ionogel	Soft	0.1<br>0.07<br>0.03<br>0.02	0.8<br>0.4<br>0.15<br>0.1	2<br>1.5<br>0.8<br>0.8	4.6<br>2<br>1.2<br>0.8	0.43<br>0.75<br>0.67<br>1.00
**S8**	hydrogel	Doubel network	0.54	1.5	1.45	5.4	0.27
**S9**	hydrogel	Doubel network	0.29<br>0.24	0.92<br>0.64	2.84<br>0.55	4<br>2.5	0.71<br>0.22
		Single network	0.05	0.18	0.1	0.4	0.25
**S10**	hydrogel	Phase separation	1<br>3<br>6<br>14<br>25	2<br>6<br>11<br>16<br>18	5<br>18<br>36<br>58<br>65	7<br>25<br>60<br>120<br>130	0.71<br>0.72<br>0.60<br>0.48<br>0.50
**S11**	hydrogel	Phase separation	2	23.5	170	180	0.94

**S12**	hydrogel	Phase separation	3	11	25	90	0.28
**S13**	hydrogel	Phase separation	2<br>0.8	2<br>1.5	4<br>1	8<br>6	0.50<br>0.17
**S14**	hydrogel	Hybrid	1100<br>420<br>12	70<br>50<br>4	7.5<br>7.5<br>6.7	3.5<br>5<br>0.6	2.14<br>1.50<br>11.17
**S15**	hydrogel	Hybrid	15.4	5.5	5.7	1.2	4.75

Supplementary Table 6. Toughness and GF values of FRCIs and recently reported gel materials. The data of gel materials were extracted from the literature.

Ref.	Materials	Structure/method	Γ (kJ m^{-2})	GF
**This work**	Ionogel	Interfacial locking	2278	784.6
**S3**	Ionogel	Supramolecular	50	9.2
**S5**	Ionogel	Composite	38	9.8
**S6**	Ionogel	Composite	85	10.12
**S7**	Ionogel	Soft	2	0.8
**S14**	Hydrogel	Hybrid	7.5	2
**S16**	Ionogel	Supramolecular	12	3.67
**S17**	Ionogel	Supramolecular	42.4	1.11
**S18**	Elastomer	Supramolecular	243	6.1
**S19**	Hydrogel	Hybrid	16.3	66.8
**S20**	Hydrogel	Phase separation	660	3.1

Supplementary References

S1 Wang, M. *et al.* Tough and Stretchable Ionogels by in situ Phase Separation. *Nat. Mater.* **21**, 359-365 (2022).

S2 Li, W. *et al.* Supramolecular Ionogels Tougher Than Metals. *Adv. Mater.* **35**, 2301383 (2023).

S3 Li, L. *et al.* Ultra-Tough and Recyclable Ionogels Constructed by Coordinated Supramolecular Solvents. *Angew. Chem. Int. Ed.* **61**, e202212512 (2022).

S4 Yao, P. *et al.* Environmentally Stable, Robust, Adhesive and Conductive Supramolecular Deep Eutectic Gels as Ultrasensitive Flexible Temperature Sensor. *Adv. Mater.* **35**, 2300114 (2023).

S5 Li, W. *et al.* Recyclable, Healable, and Tough Ionogels Insensitive to Crack Propagation. *Adv. Mater.* **34**, 2203049 (2022).

S6 Xia, Q. *et al.* Metal-organic framework (MOF) facilitated highly stretchable and fatigue-resistant ionogels for recyclable sensors. *Mater. Horiz.* **9**, 2881-2892 (2022).

S7 Yiming, B. *et al.* Ambiently and Mechanically Stable Ionogels for Soft Ionotronics. *Adv. Funct. Mater.* **31**,

2102773 (2021).

S8 Zhang, M. *et al.* Toughening Double Network Hydrogels by Polyelectrolytes. *Adv. Mater.* **35**, e2301551 (2023).

S9 Wang, Z. *et al.* Toughening hydrogels through force-triggered chemical reactions that lengthen polymer strands. *Science* **374**, 193-196 (2021).

S10 Xu, L., Qiao, Y. & Qiu, D. Coordinatively Stiffen and Toughen Hydrogels with Adaptable Crystal-domain Cross-linking. *Adv. Mater.* **35**, e2209913 (2023).

S11 Hua, M. *et al.* Strong tough hydrogels via the synergy of freeze-casting and salting out. *Nature* **590**, 594-599 (2021).

S12 Wu, Y. *et al.* Solvent-Exchange Assisted Wet-Annealing: a New Strategy for Super-Strong, Tough, Stretchable and Anti-Fatigue Hydrogels. *Adv. Mater.* **35**, e2210624 (2023).

S13 Sun, T. L. *et al.* Physical hydrogels composed of polyampholytes demonstrate high toughness and viscoelasticity. *Nat. Mater.* **12**, 932-937 (2013).

S14 Sun, M. *et al.* Multifunctional tendon-mimetic hydrogels. *Sci. Adv.* **9**, eade6973 (2023).

S15 Zhou, Q. *et al.* Mechanically Strong and Multifunctional Hybrid Hydrogels with Ultrahigh Electrical Conductivity. *Adv. Funct. Mater.* **31**, 2104536 (2021).

S16 Zhang, X. *et al.* Tough Liquid-Free Ionic Conductive Elastomers with Robust Adhesion and Self-Healing Properties for Ionotronic Devices. *Adv. Funct. Mater.* **34**, 2307400 (2024).

S17 Zhang, H., Tang, N., Yu, X., Li, M. H. & Hu, J. Strong and Tough Physical Eutectogels Regulated by the Spatiotemporal Expression of Non-Covalent Interactions. *Adv. Funct. Mater.* **32**, 2206305 (2022).

S18 Li, L. *et al.* High-Toughness and High-Strength Solvent-Free Linear Poly(ionic liquid) Elastomers. *Adv. Mater.* **35**, 2308547 (2023).

S19 Wang, J., Wu, B., Wei, P., Sun, S. & Wu, P. Fatigue-free Artificial Ionic Skin Toughened by Self-healable Elastic Nanomesh. *Nat. Commun.* **13**, 4411 (2022).

S20 Dong, X. *et al.* Strong and Tough Conductive Organo-Hydrogels via Freeze-Casting Assisted Solution Substitution. *Adv. Funct. Mater.* **32**, 2203610 (2022).
